# Hepatitis C virus NS5A protein cooperates with phosphatidylinositol 4-kinase IIIα to induce mitochondrial fragmentation

**DOI:** 10.1038/srep23464

**Published:** 2016-03-24

**Authors:** Gavin Ka Yu Siu, Fan Zhou, Mei Kuen Yu, Leiliang Zhang, Tuanlao Wang, Yongheng Liang, Yangchao Chen, Hsiao Chang Chan, Sidney Yu

**Affiliations:** 1 School of Biomedical Sciences and The Chinese University of Hong Kong, Shatin, N.T., Hong Kong SAR, P.R. China; 2 College of Life Sciences, Key Laboratory of Agricultural Environmental Microbiology of MOA, Nanjing Agricultural University, Nanjing 210095, China; 3 Epithelial Cell Biology Research Centre, The Chinese University of Hong Kong, Shatin, N.T., Hong Kong SAR, P.R. China; 4 MOH Key Laboratory of Systems Biology of Pathogens, Institute of Pathogen Biology, Chinese Academy of Medical Sciences & Peking Union Medical College, Beijing 100176, China; 5 School of Pharmaceutical Sciences, Xiamen University, Fujian 361102, China

## Abstract

Hepatitis C virus (HCV) has long been observed to take advantage of the host mitochondria to support viral replication and assembly. The HCV core protein has been implicated to fragment host mitochondria. In this report, we have discovered that the non-structural protein 5A (NS5A) plays an instructive role in attaching ER with mitochondria, causing mitochondrial fragmentation. Dynamin-related protein 1(Drp1), a host protein essential to mitochondrial membrane fission, does not play a role in NS5A-induced mitochondrial fragmentation. Instead, phosphatidylinositol 4-kinase IIIα (PI4KA), which has been demonstrated to bind to NS5A and is required to support HCV life cycle, is required for NS5A to induce mitochondrial fragmentation. Both NS5A and core are required by HCV to fragment the mitochondria, as inhibiting either of their respective downstream proteins, PI4KA or Drp1, resulted in lengthening of mitochondria tubules in HCVcc-infected cells. By fragmenting the mitochondria, NS5A renders the cells more resistant to mitochondria mediated apoptosis. This finding indicates previously-ignored contribution of NS5A in HCV-induced mitochondria dysfunction.

Hepatitis C virus (HCV) is a RNA virus affecting over 170 million people worldwide. Chronic HCV infection often leads to various liver diseases including cirrhosis and hepatocellular carcinoma. The HCV viral genome is translated in host cells as a polyprotein that is cleaved to generate 10 viral proteins. Many of these proteins have specific enzymatic activities. However, no enzymatic activity has been identified in the non-structural protein 5A (NS5A). Instead, this protein interacts with and regulates a large number of host factors to ensure efficient HCV replication and virion assembly.

Phosphatidylinositol 4-kinase IIIα (PI4KA) is one of the NS5A-interacting proteins. NS5A has a complex functional relationship with PI4KA. Depletion of PI4KA by siRNA changes the subcellular localization of NS5A and the morphology of the HCV-induced membranous web^1^,^2^. siRNA depletion or treatment with PI4KA inhibitors reduces HCV viral RNA replication. NS5A stimulates the kinase activity of PI4KA, suggesting that the kinase is a downstream effector of NS5A function^3^,^4^. NS5A is a phospho protein with 56 kd and 58 kd isoforms, for basally and hyperphosphorylated forms, respectively. PI4KA activity correlates with the abundance of basally phosphorylated form. As the status of phosphorylation affects the functions of NS5A, PI4KA is also a regulator of NS5A. Detailed interaction studies showed that residues 401 to 600 of PI4KA contain the NS5A interaction motif^5^. Overexpression of these residues confers a dominant negative effect on expression of the HCV-induced membranous web phenotype, on the amount of cellular PI4P and on HCV viral replication. Similarly, a PI4KA binding motif has been identified on NS5A. Mutating critical amino acid residues within this motif drastically reduces the interaction between the mutant NS5A and PI4KA and abrogates the stimulation of PI4KA kinase activity^6^. Although the consequence of the interaction between NS5A and PI4KA on the HCV life cycle is well-documented, the underlying downstream effect of this interaction remains to be elucidated.

HCV induces massive membrane rearrangement to form the membranous web suitable for viral replication and virion assembly. Various host membranes have been reported to contribute to forming the membranous web^2^,^7^,^8^. It has long been observed that HCV infection alters the functions of mitochondria. In cells overexpressing various HCV viral proteins, mitochondrial membrane potential is altered and oxidative phosphorylation is drastically compromised, yet an increase in ATP production was also observed^9^,^10^. This may be due to a shift of energy production toward glycolysis. HCV viral proteins induce the transcription of genes encoding glycolytic enzymes via Hypoxia-Inducible Factor 1α^9^. In this study, several viral proteins including NS5A were reported to be associated with mitochondria but the effect on mitochondria dysfunction is attributed to the core protein. Liver mitochondria isolated from transgenic mice expressing the HCV core protein have increased production of reactive oxygen species (ROS) and calcium uptake by mitochondria^11^. The effect of HCV on mitochondria is not limited to metabolism. HCV inactivates host innate immunity by NS3/4A-mediated cleavage of mitochondria-bound MAVS, an important signaling molecule in the induction of interferons^12^. MAVS cleavage takes place in a specialized region of the mitochondria with attachment to ER membranes, called the mitochondria-attached ER membranes (MAM)^13^. HCV infection has also been reported to stimulate mitophagy by promoting the translocation of mitophagy marker protein Parkin to mitochondria^14^, to inhibit mitochondria-mediated apoptosis^15^, and to cause mitochondria fission^15^. Precisely which viral proteins are responsible for these effects remain unknown but the core appears to play very important role.

In morphological studies of the HCV-induced membranous web using fluorescence and electron microscopy, we unexpectedly discovered that the NS5A protein plays an instructive role in tethering the membranes between endoplasmic reticulum and mitochondria, leading to fragmentation of the mitochondria. This function is not dependent on dynamin related protein 1 (Drp1), but on PI4KA.

## Results

### ER-mitochondria tethering in HCV replicon cells

**Figure 1:**
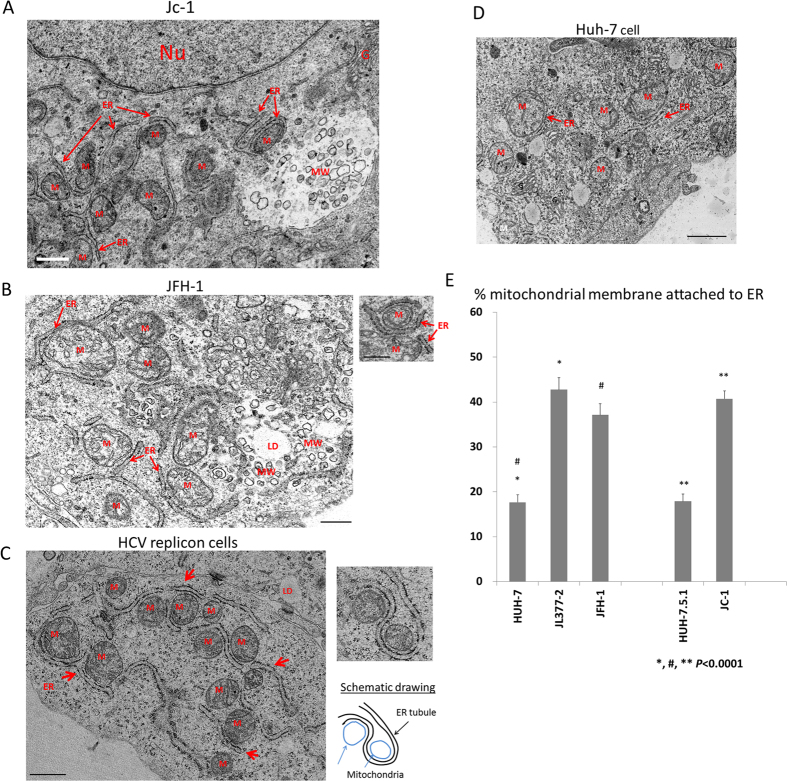
Extensive attachment of ER tubules to mitochondria in HCV replicon cells JL377-2 and hepatocytes transfected with JFH-1 or infected with HCVcc. (**A**) Electron micrographs of Huh-7.5.1 cells infected with HCVcc. Scale bar = 0.5 μm. (**B**) EM images of Huh-7 cells transfected with JFH-1 full-length HCV genomic RNA. Scale bar = 0.5 μm. (**C**) EM images of the cytoplasm of a JL377-2 cell. Scale bar = 1μm. (**D**) EM image of naïve Huh-7 cells (left panel). Scale bar = 1μm. Abbreviations: M, mitochondria; ER, endoplasmic reticulum; G, Golgi; Nu, nucleus; MW, membranous web, LD, lipid droplet. (**E**) Quantification of the amount of mitochondria membrane tethered with ER. The percentage of perimeter membrane of mitochondria closely apposed to ER tubules in EM images of the indicated HCV harboring cells or naïve Huh-7 or Huh-7.5.1 cells were quantified. N = 60; error bars = S.E.M.; p < 0.0001.

Electron micrograph (EM) images of Huh-7.5.1 cells infected with HCVcc or Huh-7 cells transfected with JFH-1 full-length HCV genomic RNA showed extensive tethering between the ER and the mitochondria (Fig. 1A,B, respectively), with the space between the membranes estimated to be 20 nm or less. In one occasion, an ER tubule completely encircling a mitochondrion was observed (Fig. 1B inset). It has been well-documented that ER and mitochondria form contact sites called MAM for exchange of calcium, lipids, and other functions such as mitochondrial fission and inflammasome formation^16^,^17^. We initially suspected the HCV core protein was responsible for the ER-mitochondria tethering effect but this phenomenon was also extensively observed in EM images of Huh-7 cells harboring sub-genomic HCV replicon (JL-377-2 cells), in which core was absent (Fig. 1C). Highly curved ER tubule wrapping around two mitochondria in an “S” shape was observed on one occasion and a schematic drawing of the structures is presented (Fig. 1C). ER-mitochondria tethered membrane can also be found in Huh-7 cells not expressing any HCV proteins but is much less frequently observed (Fig. 1D). We carefully quantified the amount of mitochondrial membrane tethered with ER tubules in EM images of hepatocytes carrying different types of HCV genomes and in naïve cells and found that HCV replicon, HCV transfection, and HCVcc infection all caused over two-fold increase in mitochondria membrane tethered by ER tubules. Approximately 40% of the mitochondrial membrane in cells containing HCV is attached to ER, compared to less than 20% in corresponding naïve hepatocytes (Fig. 1E). Because ER-mitochondria tethering was also drastically increased in cells expressing the HCV replicon, which lacks the structural genes, we suspect at least one of the non-structural proteins may have such function.

### NS5A tethers ER-mitochondria and causes mitochondrial fragmentation

**Figure 2:**
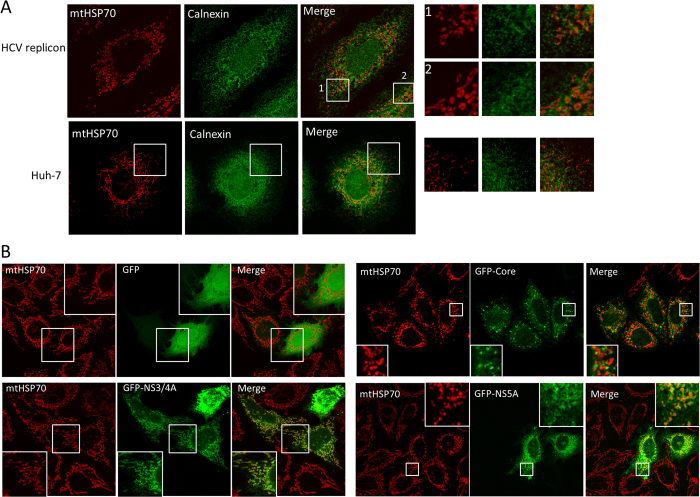
NS5A and NS3/4A play a role in tethering ER and mitochondria. (**A**) Immunofluorescence images of HCV replicon cells JL-377-2 and naïve Huh-7 cells staining with mitochondria marker mtHSP70 (red) and ER marker Calnexin (green). (**B**) GFP (top row of panels), GFP-NS3/4A (second row), GFP-core (third row) and GFP-NS5A (bottom row) were expressed in HEK293 cells and tested for their ability to attach to mitochondria (mtHSP70).

Using confocal microscopy, we confirmed that ER membrane was closely associated with mitochondria membrane in cells expressing the HCV replicon (top panels, Fig. 2A). In this experiment, a mitochondria-specific heat-shock protein 70 was used as marker for mitochondria (red), and calnexin was used as ER marker (green). The majority of the mitochondria signal in cells expressing the HCV replicon was highly fragmented (inset 1), and was surrounded by ER signal (inset 2). In contrast, mitochondria from naïve Huh-7 cells was largely short tubules that were poorly colocalized with ER signals (bottom panels and inset, Fig. 2A).

Next, we took a candidate gene approach to identify which viral protein(s) within the HCV replicon cells conferred the ability to tether ER and mitochondria, using HEK293 cells for their high transfection efficiency and long mitochondria tubules where ER-tethering and mitochondrial fragmentation could easily be observed. Furthermore, since HEK293 cells cannot support HCV replication, any effects caused by expressing the HCV viral proteins are likely to be intrinsic biochemical properties of the viral protein in question. A similar investigation of the cleavage of MAVS on mitochondria by NS3/4A was conducted in HEK293 cells^12^,^13^. When we overexpressed NS3/4A in HEK293 cells, GFP-NS3/4A showed a fluorescence pattern similar to ER that surrounded the mitochondria marker mtHSP70 (bottom left panels, Fig. 2B), suggesting that NS3/4A could be the viral proteins responsible for tethering ER and mitochondria. The core protein has been reported to regulate host mitophagy and alter various mitochondrial functions^9^,^10^,^11^,^14^, but when we overexpressed GFP-core fusion protein, we only observed partial colocalization between GFP-core and mitochondria (top right panels, Fig. 2B). The mitochondria in the GFP-core transfected cells were fragmented, confirming an action of the core protein on mitochondria. Surprisingly, when HEK293 cells were transfected with GFP-NS5A, the mitochondria was fragmented into mostly dot-like membrane structures, extensively surrounded by GFP-NS5A signal similar to the effect of NS3/4A (bottom right panels, Fig. 2B). In this experiment, GFP overexpression did not alter mitochondrial morphology (top left panels, Fig. 2B). These results not only confirm previously documented functions of NS3/4A and core on mitochondria, but also indicate a novel effect of NS5A in attaching ER and mitochondria membranes. The ER-mitochondria tethering effect of NS5A was also observed in other commonly used cell lines such as COS, suggesting NS5A has intrinsic biochemical activity that is independent on the cellular context (data not shown).

**Figure 3:**
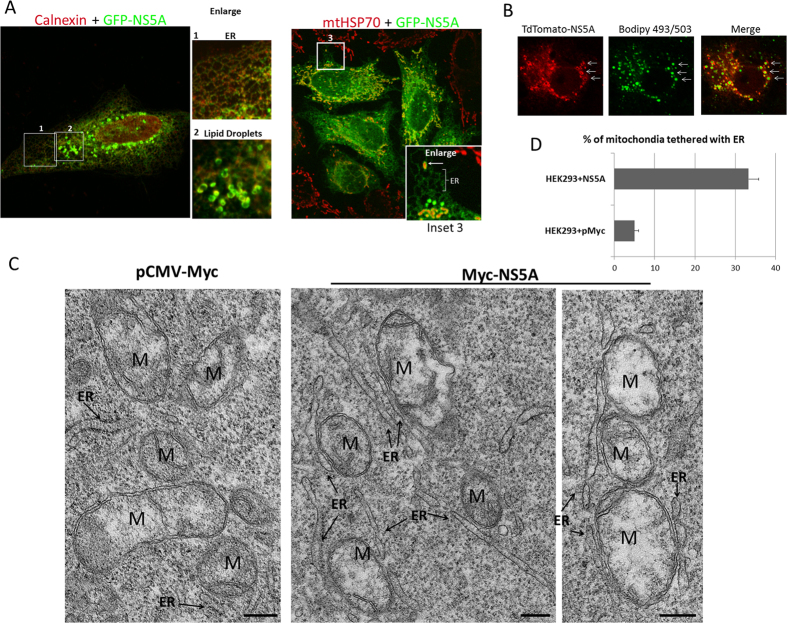
NS5A expression in HEK293 cells and Huh-7 cells. (**A**) GFP-NS5A is localized to ER membranes (inset 1 and bracket, inset 3) and induces formation of lipid droplets (inset 2). GFP-NS5A frequently fragments and encircles mitochondria (arrow, inset 3). In this experiment, HEK293 cells were transfected with GFP-NS5A for 18 hours before fixation and staining with mtHSP70 (red). (**B**) TdTomato-NS5A (red) transfected into Huh-7 cells and stained with lipid droplet marker bodipy 493/503 (green). NS5A encircles lipid droplets (arrows). (**C**) EM study of the effect of NS5A in ER-mitochondria tethering in HEK293 cells transfected with Myc-NS5A or pMyc vector. Extensive ER-tethered mitochondria was observed in Myc-NS5A transfected HEK293 cells but not in Myc vector transfected cells. Scale bars = 1 μm. Abbreviations: M, mitochondria; ER, endoplasmic reticulum. (**D**) Quantification of ER-mitochondria tethering in NS5A-transfected HEK293 cells from EM images. The percentage of ER-tethered mitochondria is determined by the length of the mitochondria perimeter apposed to ER tubules in the EM images. N = 60; error bar = S.E.M.

GFP-NS5A retained its ER localization in HEK293 cells, (Fig. 3A, inset 1) and was capable of inducing membrane structures that are likely lipid droplets (Fig. 3A, inset 2). To confirm these structures were lipid droplets, we expressed a TdTomato-NS5A in Huh-7 cells, which can efficiently take up lipids and form larger lipid droplets, and then labeled these lipid droplets with bodipy 493/503. The NS5A-induced membrane structures surrounded the bodipy 493/503-labelled lipid droplets (arrows, Fig. 3B). Similar result, except with weaker staining intensity of bodipy 493/503, was also observed in HEK293 cells (data not shown). These results suggested the expression of NS5A in HEK293 cells was not substantially different from its expression in hepatocytes and most of its functions were preserved in HEK293. More importantly, NS5A signal could extensively encircle the mitochondria (Fig. 3A and inset 3, arrow). We confirmed that ER-mitochondria tethering was induced by NS5A by performing EM studies on NS5A transfected HEK293 cells (Fig. 3C). Extensive ER-tethered mitochondria membrane was observed in HEK293 cells transfected with Myc-NS5A but not pMyc vector. Using the same quantitative method as in Fig. 1, we determined that on average in the former, approximately 32% of the mitochondria membrane surface was attached to ER membrane, compared to about 5% in the vector-transfected control (Fig. 3D). Therefore, ER-localized NS5A could induce the formation of lipid droplets from ER membrane and tethered ER and mitochondria.

**Figure 4:**
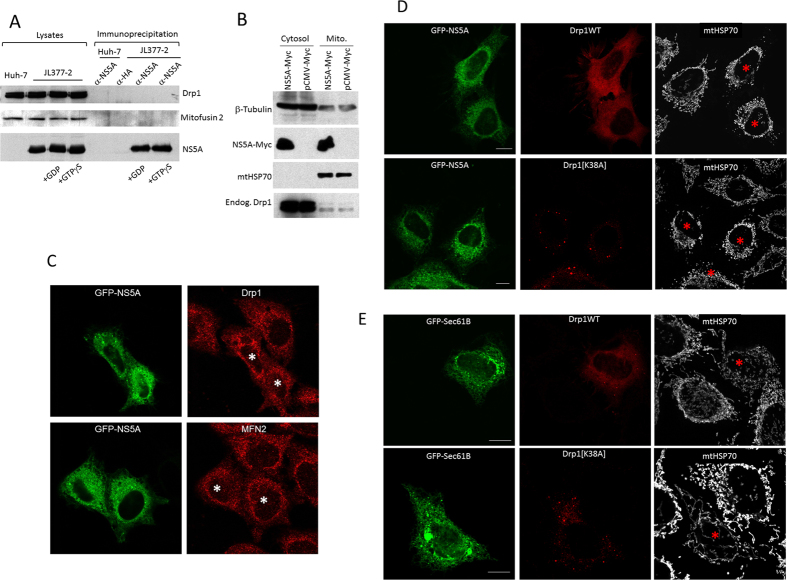
NS5A-induced mitochondrial fragmentation is independent of Drp1. (**A**) NS5A does not interact with Drp1. Huh-7 or JL377-2 cell lysates were subjected to immunoprecipitation by α-NS5A antibody in the presence of GDP or GTPγS. A monoclonal antibody against HA was used as a control. In all conditions, neither endogenous Drp1 nor mitofusin 2 was co-precipitated with NS5A. (**B**) NS5A does not increase the mitochondrial association of Drp1. HEK293 cells overexpressed with Myc-NS5A or control Myc-empty vector and the extent of Drp1 on mitochondria-enriched membranes were determined by immunoblotting. (**C**) Overexpression of GFP-NS5A does not change the subcellular localization of endogenous Drp1 and mitofusin 2 (MFN2) by immunofluorescence. GFP-NS5A expressing cells are marked with asterisks. (**D**) Wildtype Drp1 or Drp1[K38A] dominant negative mutant cDNA sequences (red) were cotransfected with GFP-NS5A (green) into HEK293 cells. The mitochondria were visualized by staining the cells with mtHSP70 at 633 nm wavelength excitation laser (white). (**E**) Drp1 or Drp1[K38A] dominant negative mutant cDNA sequences (red) were cotransfected with control GFP-Sec61B (green) into HEK293 cells. The mitochondria were visualized by staining the cells with mtHSP70 at 633 nm wavelength excitation laser (white). Red asterisks indicate transfected cells. Scale Bar = 10 μm.

Since HCV is reported to fragment the mitochondria in a Drp1-dependent pathway, we investigated whether host factors important in mitochondria fission and fusion were required for NS5A-induced mitochondrial fragmentation. Two such molecules are Drp1 and mitofusin 2, important in mediating mitochondria fission and fusion, respectively. As shown in Fig. 4A, NS5A did not interact with Drp1 or mitofusin2 when co-transfected into HEK293 cells (Fig. 4A), regardless of the status of guanine nucleotide binding of Drp1. Further, NS5A overexpression did not redistribute more Drp1 to mitochondria-enriched membrane fraction (Fig. 4B). Immunofluorescence staining of endogenous Drp1 and mitofusin 2 in NS5A-transfected HEK293 cells revealed no change in the subcellular localization of these proteins (Fig. 4C). When NS5A was co-transfected with the dominant negative mutant of Drp1 into HEK293 cells, the mitochondria of the transfected cells were still highly fragmented (top panels, Fig. 4D). In comparison, GFP-Sec61B co-transfected with Drp1 resulted in aberrantly long and tubular mitochondria (bottom panels, Fig. 4E). GFP-Sec61B is a GFP-ER marker. As the mitochondrial fragmentation induced by NS5A was not suppressed by the dominant negative effect of the Drp1 mutant, NS5A must fragment the mitochondria by a Drp1-independent mechanism.

### NS5A cooperates with PI4KA to mediate mitochondrial fragmentation

**Figure 5:**
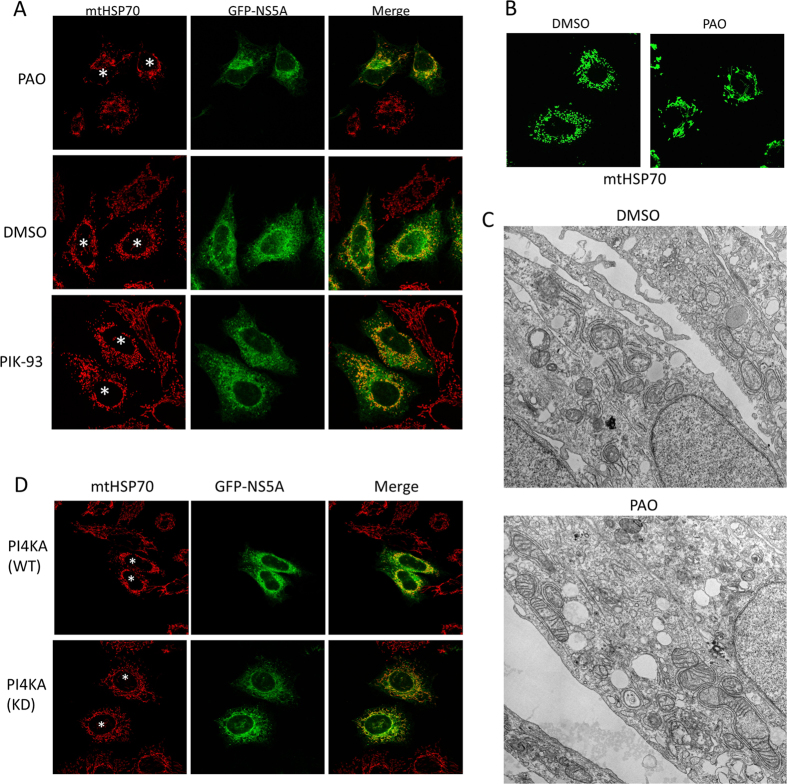
PI4KA is required for NS5A to cause mitochondrial fragmentation. (**A**) PI4KA inhibitor PAO but not PI4KB inhibitor PIK-93 or vehicle DMSO modulates the effect of NS5A on mitochondrial fragmentation. HEK293 cells transfected with GFP-NS5A were treated with the indicated inhibitors before immunofluorescence staining. Asterisks indicate the mitochondria of GFP-NS5A transfected cells. (**B**) JL377-2 HCV replicon cells treated with PAO were stained with mitochondria marker mtHSP70. (**C**) EM ultrastructures of JL377-2 cells treated with DMSO or PAO. (**D**) Co-expression of the kinase deficient mutant of PI4KA modulates the mitochondrial fragmentation caused by GFP-NS5A in HEK293 cells. GFP-NS5A and PI4KA DNA constructs were transfected at ratio of 1:5 into HEK293 cells. The cells were subjected to immunofluorescence staining using antibody against mtHSP70 to label the mitochondria (red). Asterisks mark the cells expressing the indicating DNA constructs.

NS5A interacts with a number of cellular proteins and affects many signaling pathways. We investigated if its function in fragmenting the ER-tethered mitochondria required some of the NS5A-interacting proteins that were likely to regulate host membranes^3^,^4^,^5^,^18^,^19^,^20^,^21^,^22^,^23^,^24^. We co-transfected NS5A with cDNAs of these genes, including VAP-B, PI4KA, PI4KB, amphiphysin II, Rab1, TBC1D20, c-Src, or treated the NS5A-transfected cells with small inhibitory compounds specific to some of the host proteins. PI4KIIIα (PI4KA), when inhibited, consistently alleviated the NS5A-induced mitochondrial fragmentation. Reportedly, PI4KIIIα (PI4KA), but not PI4KIIIβ (PI4KB), interacts with NS5A^24^, although both kinases are apparently required to support HCV replication. We tested if specific kinase inhibitors to these lipid kinases could reverse fragmentation of mitochondrial fragmentation induced by overexpressed NS5A. In HEK293 cells incubated with phenylarsine oxide (PAO), which inhibits PI4KA, mitochondria largely became tubular again (Fig. 5A, top panels). In comparison, PI4KB inhibitor, PIK-93, or vehicle solvent DMSO could not reverse the mitochondrial fragmentation phenotype (cells marked with asterisks, middle and bottom panels, Fig. 5A). Therefore, PI4KA appeared to cooperate with NS5A to fragment mitochondria. The effect by PAO can be recapitulated in cells trafected with the HCV replicon (Fig. 5B) or infected with HCVcc (below), as mitochondrial tubules started to appear one hour after PAO was applied to the cells. EM images showed that treating JL377-2 cells with PAO increased the size of the mitochondria even though these mitochondria were still highly attached with ER tubules (Fig. 5C).We obtained cDNA encoding PI4KA and its kinase deficient mutant (KD), co-expressed these molecules with NS5A and determined the status of the mitochondria in HEK293 cells. Co-expression of wildtype PI4KA and NS5A caused mitochondrial fragmentation (top panels, Fig. 5D), but the PI4KA(KD) mutant completely reversed the mitochondrial fragmentation effect of NS5A (bottom panels, Fig. 5D). PI4KA or its KD mutant, when co-expressed with control GFP-Sec61B, did not cause morphological change to the mitochondria (data not shown), suggesting that endogenous PI4KA does not normally affect mitochondria morphology.

**Figure 6:**
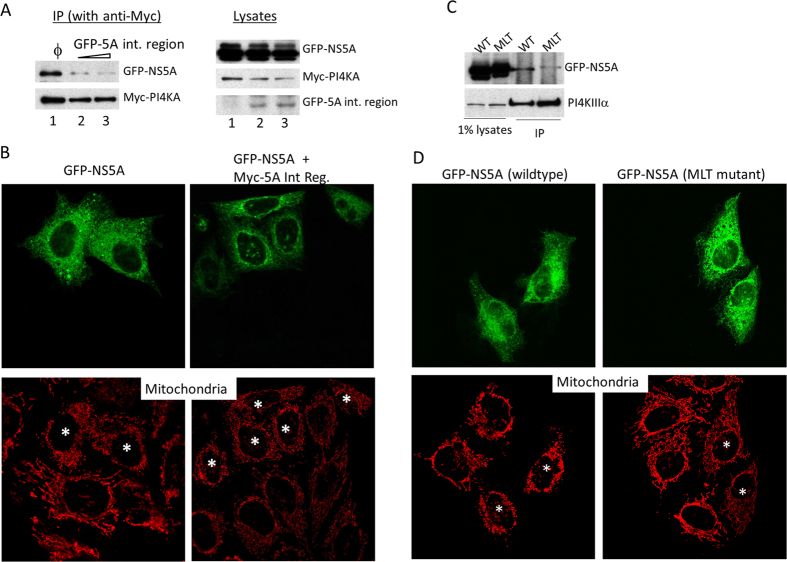
Interaction between NS5A and PI4KA is critical for the NS5A-induced mitochondrial fragmentation. (**A**) Disruption of the NS5A-PI4KA interaction by overexpression the NS5A-interacting region of PI4KA in HEK293T cells. The protein interaction between GFP-NS5A and Myc-PI4KA was investigated by immunoblotting after immunoprecipitation using anti-Myc antibody. The interaction between GFP-NS5A and Myc-PI4KA can be disrupted by co-expression of GFP-5A int. region (lanes 2 and 3). (**B**) Disruption of NS5A-PI4KA interaction can abrogate the mitochondrial fragmentation activity of NS5A. GFP-NS5A alone or in combination with Myc-5A interacting region (Myc-5A Int Reg.) at a ratio of 1:5 was transfected into HEK293 cells which were subsequently stained with mitochondria marker mtHSP70. Transfected cells are indicated with asterisks. (**C**) NS5A with mutations at the PI4KA binding motif (MLT mutant) shows significantly reduced binding to PI4KA. GFP-NS5A wildtype (WT) or MLT mutant (MLT) were co-expressed with Myc-PI4KA in HEK293T cells and immunoprecipitated with anti-Myc 9E10 antibody (bottom panel). The extent of co-precipitated GFP-NS5A proteins was investigated by immunoblotting (top panel). (**D**) Overexpression of the NS5A MLT mutant no longer caused mitochondrial fragmentation. GFP-NS5A wildtype or MLT mutant were expressed in HEK293 cells (top panels) and the status of mitochondria was analyzed with immunofluorescence staining of mtHSP70 (bottom panels). Asterisks indicate the corresponding NS5A transfected cells.

PI4KA binds to NS5A using the domain encompassing residues 401 to 600 (the NS5A-interacting region or 5A-int. region)^5^. Conversely, the PI4KA-interaction motif on NS5A was mapped to a stretch of amino acids from residues 187 to 214^6^. When critical amino acids within this region were mutated to alanine, the mutant NS5A binds poorly to PI4KA. One such mutant has triple alanines replacing the MLT residues at the PI4KA binding site^6^. Overexpressing the 5A-int. region inhibited the interaction between full-length PI4KA protein and NS5A as measured by co-immunoprecipitation (Fig. 6A). When the 5A-int. region was co-expressed with GFP-NS5A, mitochondrial fragmentation was abrogated (Fig. 6B, asterisks), and the tubular pattern of the mitochondria in transfected cells was no different from the nearby non-transfected cells. We constructed a mutant NS5A that interacted poorly with PI4KA by changing the MLT residues to alanine residues. This mutant (indicated as MLT), bound very poorly to PI4KA by co-immunoprecipitation experiment (Fig. 6C). When overexpressed this mutant failed to fragment mitochondria, even though the mutant was properly localized to ER membranes (Fig. 6D). Together, these results suggest that the interaction between PI4KA and NS5A is critical for inducing mitochondrial fragmentation.

**Figure 7:**
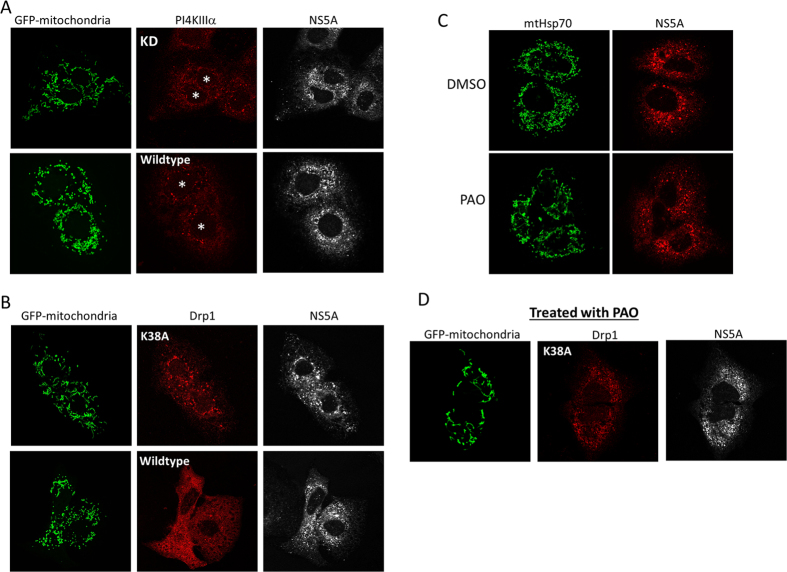
Drp1 and PI4KA are both necessary in mitochondrial fragmentation in HCV infected cells. (**A**) Mitochondrial fragmentation is reduced by the kinase deficient (KD) mutant of PI4KA in Huh-7.5.1 cells infected with HCVcc (Jc-1). Asterisks indicate PI4KA transfected cells. NS5A staining indicates HCV infected cells. (**B**) Mitochondrial fragmentation is reduced by dominant negative Drp1 mutant (K38A) in HCVcc-infected cells. (**C**) PI4KA inhibitor PAO reduces mitochondrial fragmentation in Huh-7.5 cells infected with HCVcc. (**D**) Treating HCVcc infected cells that have been transfected with Drp1 (K38A) with PAO did not cause drastic increase in mitochondria tubular length.

Because the core-Drp1 interaction and NS5A-PI4KA interaction are both capable of fragmenting the mitochondria, we wanted to learn which interaction was more physiologically relevant in HCV infection. Huh-7.5.1 cells infected with HCV, which express both core and NS5A, were transfected with plasmids encoding the dominant negative mutant of Drp1 (K38A) or the kinase deficient mutant of PI4KA (KD). As shown in Fig. 7A,B, short tubules of mitochondria appeared when either plasmids encoding Drp1(K38A) or PI4KA(KD) were transfected into the cells. This result suggests that at protein expression level that supports HCV life cycle, both core and NS5A contribute to the fragmentation of the host mitochondria. Furthermore, short mitochondria tubules started to appear in HCVcc-infected cells upon inhibiting PI4KA with PAO (Fig. 7C). We investigated if inhibiting both Drp1 and PI4KA would further increase the mitochondria tubular length by transfecting Huh-7.5.1 cells with the DRP1(K38A) plasmid and then treating with PAO, but did not find aberrantly long mitochondria tubules from this treatment (Fig. 7D). Rather, clustering of the mitochondria was obvious (Fig. 7D), and sometimes such clustering happened around the perinuclear region (data not shown).

### NS5A increases resistance to mitochondria-mediated apoptosis

**Figure 8:**
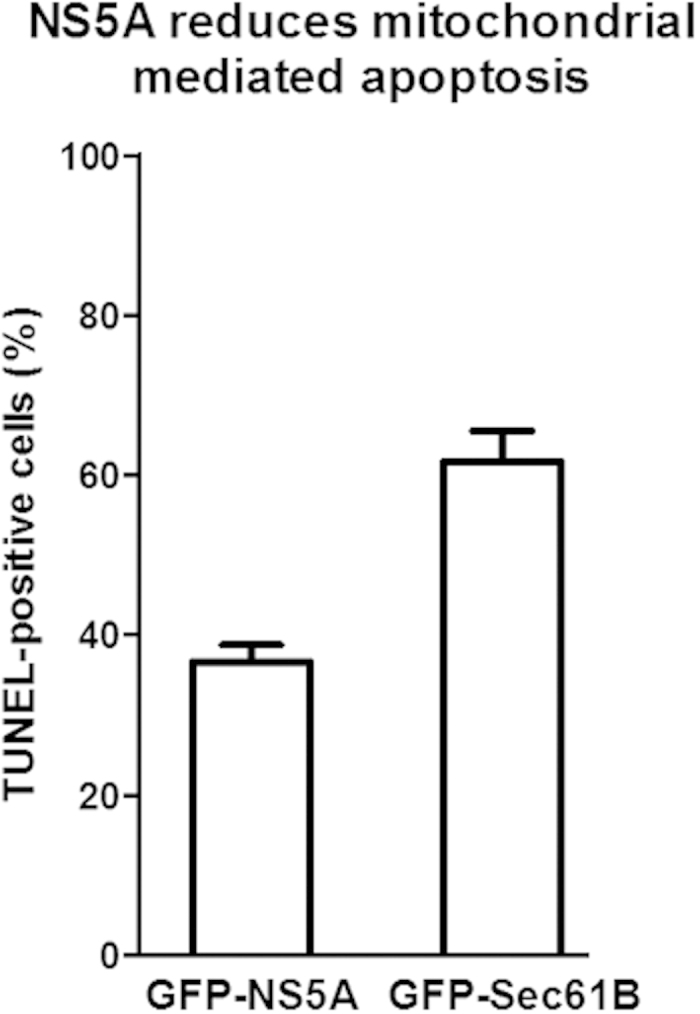
NS5A reduces mitochondrial mediated apoptosis. GFP-NS5A or control GFP-Sec61B transfected HEK293 cells were treated with hydrogen peroxide before TUNEL staining for detection of apoptotic cells. In five independent experiments, a total of over 500 transfected cells were counted in each condition; error bars = S.E.M.; P < 0.0001.

To determine the functional consequence of NS5A-induced mitochondrial fragmentation, we investigated if mitochondria-mediated apoptosis was affected. When transfected HEK293 cells expressing GFP-NS5A were treated with hydrogen peroxide to induce apoptosis, the percentage of cells undergoing apoptosis was significantly reduced (36.7%), compared to transfected control cells expressing GFP-Sec61B (61.6%) (Fig. 8). Therefore, a functional consequence of the fragmentation of mitochondria by NS5A was an increase in resistance to apoptosis.

## Discussion

Previously electron microscopy revealed extensive ER-mitochondria tethering in cells expressing a HCV replicon but little in naïve Huh-7 cells^25^. However, HCV-induced ER-mitochondrial tethering was not investigated in this and subsequent EM studies on other HCV contexts^2^,^15^. Here, we have extensively investigated the ER-mitochondria tethering induced by HCV, and identified NS5A and NS3/4A as being two viral proteins capable of such biochemical activity, and have observed that NS5A extensively fragments mitochondria.

Mitochondria are affected during HCV pathogenesis in several aspects including alteration of mitochondrial membrane potential, increase in reactive oxygen species, in calcium uptake, in energy production via glycolysis, in mitophagy and cleavage of inflammatory signaling molecules^9^,^10^,^11^,^13^,^14^,^26^,^27^ . In most cases, the HCV core protein and NS3/4A are involved in alteration of mitochondrial functions and mitochondria-bound inflammatory signaling. However, when overexpressed in HEK293 cells, core protein partially localized to mitochondria. ER-localized NS3/4A tethers the mitochondria extensively but its effect on mitochondrial fragmentation is not strong. HCV core protein fragments the mitochondria but is not highly enriched in ER. In this report, we used EM and fluorescence microscopy to show that ER-bound NS5A is tethered to mitochondria. Its ability to fragment mitochondria tubules was the strongest among the viral proteins tested here. This effect required intact PI4KA kinase activity and was highly dependent on the interaction between NS5A and PI4KA. By fragmenting the mitochondria, NS5A may compartmentalize the organelle and decrease the possibility of full-blown cytochrome c release from mitochondria to execute apoptosis. In addition to apoptosis, we speculate that fragmenting the mitochondria allows NS5A to increase the attached surface area between ER and mitochondria. This may accelerate processes such as mitophagy and inactivation of RIG-I signalling mediated by core and NS3/4A, respectively. NS5A is thought to be a master regulator in HCV life cycle^28^. In this regard, its ER-mitochondria tethering effect reinforces this concept: By bringing together these membranes, NS5A may allow core and NS3/4A to perform their specific, mitochondrial-related functions. The function of NS5A, however, seems limited to tethering ER and mitochondria and subsequent mitochondrial fragmentation in this context. As the cleavage of MAVS happens at the MAM, we initially speculated that NS5A may increase the conversion of ER-mitochondria attached membranes to MAM. However, this does not appear to be the case as we did not find increased signal of a MAM marker on ER-mitochondria tethered membranes induced by NS5A(data not shown).The exact functional relationship between NS5A, core and NS3/4A on mitochondria awaits further investigation.

While the interaction between PI4KA and NS5A and the effect of PI4KA on HCV replication have been well documented, the precise downstream functions of PI4KA, however, remained obscure. It has been postulated that the lipid product of PI4KA, PI(4)P, may change the phospholipid composition of the HCV-induced membranous web, so that other cellular proteins, such as oxysterol binding protein (OSBP) and VAMP associated proteins (VAP-A,B,C) can associate and function^28^. Not mutually exclusive, here we demonstrate that mitochondrial fragmentation is one of the downstream effects of the PI4KA-NS5A interaction. PI4KA has been previously regarded as normally localized to the ER and when hijacked and stimulated by NS5A, induces mitochondrial fragmentation. However, an earlier study found PI4KA in the mitochondria outer membrane by immuno-EM^29^,^30^. NS5A might simply activate the pool of mitochondria-bound PI4KA to fragment the mitochondria. In addition, it was intuitive to hypothesize that PI(4)P, the product of PI4KA, or its downstream metabolites such as phosphatidylinositol (4,5)-bisphosphate (PI(4,5)P_2_), would play a role in mitochondria fission. PI(4,5)P_2_ facilitates the activities of F -actin and Drp1 in mitochondria fission^31^. Drp1 and mitochondria fission factor (Mff) have recently been demonstrated to take part in HCV-induced mitochondria fission^15^. We suspected this observation could explain how NS5A fragments mitochondria . However, we found that neither Drp1 nor mitofusin 2 interacted with NS5A and the subcellular localizations of neither proteins was affected by NS5A (Fig. 4). Most importantly, overexpression of a dominant negative mutant of Drp1 did not block NS5A-mediated mitochondrial fragmentation (Figs 4 and 7). Therefore, the mitochondrial fragmentation mediated by NS5A is not related the classic mechanism involving Drp1 and mitofusin. However, in cells infected with HCVcc, inhibiting either Drp1 or PI4KA reduced the extent of mitochondria fragmentation, suggesting that HCV-induced mitochondrial fragmentation requires both NS5A and core. While Drp1 is not related to NS5A-induced mitochondrial fragmentation, actin-depolymerizing agent cytochalasin D can partially rescue the mitochondria from NS5A-induced fragmentation (data not shown). Since actin dynamics are heavily regulated by phospholipids including products of PI4KA, we believe the function of PI4KA in mitochondrial fragmentation must be related to actin. Determining precisely how NS5A fragments the mitochondria via PI4KA may require additional experiments. It is, however, clear that the data presented here assigns a role of PI4KA in stimulating the fission of mitochondria, and warrants re-examination of the overall function of PI4KA in a mitochondria related context. Likewise, understanding the roles of NS5A in the HCV-induced mitochondria dysfunctions may uncover aspects of HCV pathogenesis that have been previously ignored.

## Methods

### Plasmids

The nucleotide sequence encoding hepatitis C virus (HCV) non-structural 5A protein (NS5A) was obtained from the plasmid pCMV-Tag1-NS5A constructed by Budhu *et al.*^32^ (Addgene number 17646, HCV sub-genotype 1b, isolate H77) and cloned into pCMV-EGFP vector for immunofluorescence studies. GFP-NS3/4A was cloned from APP248 pCon1FL plasmid (Apath LLC, HCV sub-genotype 1b). GFP-core was cloned from CMV-FLAG-Core-R (pMO29) plasmid (Addgene number 24480, HCV sub-genotype 1b). pmCherry-Drp1 was obtained from (Addgene 49152). pCDNA-Drp1[K38A] mutant was obtained from Dr. Qinfeng Shen (UCLA). PI4KA (WT)-Myc and its corresponding kinase-inactivated mutant, PI4KA(KD)-HA were described in Li *et al.*^33^. PI4KA(KD) contains the mutation D1899A^24^,^34^. The sequences encoding the region of PI4KA (401–600 amino acids) interacting with NS5A was extracted and cloned into pCMV-Myc for the PI4KA competition study. The PI4KA MLT mutant was constructed according to the sequence information from Reiss *et al.*^6^.

### Antibodies

Endogenous NS5A was detected by a mouse monoclonal antibody from Millipore (MAB8694). Drp1 antibody was purchased from BD Biosciences (611112). For co-localization studies, antibodies of several organelle markers were being used. Mitochondrial heat shock protein 70 kDa (mtHSP70) was used as a marker for mitochondria and was detected by mouse monoclonal antibody JG1 (Abcam, AB2799). Calnexin was used as an endoplasmic reticulum (ER) marker and detected with rabbit polyclonal antibodies from Sigma (C4731). PI4KA protein was detected by rabbit polyclonal antibody from Cell Signaling (4902S). Rabbit antibodies against mitofusin 2 (MFN-2) (Cat No. AAS20424C) were purchased from Antibody Verify (USA).

All the secondary antibodies conjugated with fluorophores for immunofluorescence studies were purchase from Molecular Probes. These were Alexa Fluor 488 (A-11017), 568 (A-11019), 635 (A-31575) conjugated goat anti-mouse and 488 (A-11070), 568 (A-21069) conjugated goat anti-rabbit IgG (H+L) antibodies.

### Chemicals

Mito-Tracker Orange CM-H2TMRos (Molecular Probes, Cat No. M-7511) was supplied by Life Technologies. PI4KIIIα inhibitor, phenylarsine oxide (PAO, Sigma, Cat No. P-3075) and PI4KIIIβ inhibitor, PIK-93 (AdooQ BioScience, Cat No. A10731), were acquired from Sigma and AdooQ BioScience, respectively. Transfection reagents, polyethylenimine (PEI, Sigma Cat No. 408727) and GenJet *in vitro* DNA tranfection reagent for Huh-7 Cells (SignaGen, Cat No. SL100489-HUH) were purchased from Sigma and SignaGen, respectively. Hydrogen peroxide (Cat No. 1100066) was purchased from International Laboratory, USA). Apoptotic cells were detected by by TUNEL staining using *In situ* cell death detection kit, TMR red (Roche, Cat no. 12156792910).

For sample preparation for electron microscopy (EM), fixatives glutaldehyde, EM Grade (Electron Microscopy Sciences, Cat No. 16200) and osmium tetroxide (Electron Microscopy Sciences, Cat No. 19110); embedding medium, Embed-812 embedding kit (Electron Microscopy Sciences, Cat No. 14120); and stains, uranyl acetate (Electron Microscopy Sciences, Cat No. 22400) and lead citrate (Electron Microscopy Sciences, Cat No. 17800) were purchased from Electron Microscopy Sciences.

### Cell lines, culture conditions and viruses

HEK-293 (ATCC Cat No. CRL-1573) and JL 377-2 cell lines (ATCC Cat No. PTA-4583, Huh-7 cells containing an HCV replicon I377/NS3-3′UTR, sub-genotype 1b, isolate Con1, Accession No. AJ242652) were acquired from ATCC^35^. pJFH-1 (genotype 2a) was originally from Takaji Wakita^36^. Huh-7 cells were a gift from Dr. Ayano Satoh (Okayama University, Japan). Huh-7.5.1 cells were grown in Dulbecco’s modified Eagle’s medium (DMEM) supplemented with 10% fetal bovine serum (FBS) and incubated in 5% CO_2_ environment at 37^o^ C^33^.

To produce HCV from cells transfected with JFH-1, JFH-1 plasmid was first lineralized with Xbal restriction digestion and then transcribed *in vitro* with T7 RiboMax express large scale RNA production system (Cat No. P1320, Promega, USA) following the manufacturer’s instructions. The single strand RNA products were further purified with RNeasy Mini Kit (Cat No. 74104, Qiagen, USA) before use.

Huh-7 cells (4 × 106 cells) were mixed with 30 μg single strand RNA (ssRNA) of JFH-1 in Cytomix buffer (120 mM KCl; 0.15 mM CaCl_2_; 10 mM K_2_HPO_4_/KH2PO_4_, p.H. = 7.6, 2 mM EGTA, 5 mM MgCl_2_, 2 mM ATP, 5 mM Glutathione, 2.5 mM HEPES, pH = 7.6). Electroporation was done using Bio-Rad Gene Pulser Xcell (260 V, 1000 μF, ∞Ω, 4 mm cuvette, exponential wave form). Three days later, cells were then used for immunofluorescent and electron microscopic studies.

HCVcc was generated by method previously described^33^. In brief, naïve Huh-7.5.1 cells were seeded 24 h before infection. They were then infected with Jc-1 HCV (Jc1FLAG2(p7-nsGluc2A) expressing Gaussia luciferase, sub-genotype 2a, obtained from Charles Rice) containing medium at a multiplicity of infection (MOI) of 1. Three days later, cells were used for immunofluorescence and electron microscopic studies after checking for successful infection with a luciferase assay.

Mito-Tracker was used at 0.8 μM concentration and the cells were labeled for 45 minutes before fixation. For experiments using PAO or PIK-93, PAO (300 nM) and an equal amount of DMSO was applied to cells for 1 hour before immunofluorescence staining. PIK-93 (0.5 μM) was applied to cells for 6 hours before processing. Apoptosis was induced by treating cells with 1 mM hydrogen peroxide in serum free DMEM for 16 hours followed by TUNEL staining.

### Indirect immunofluorescence microscopy

Immunofluorescence studies were performed similarly to methods previously described^37^. Briefly, cells were seeded on 12 mm coverglass in 4-well plate before any treatments. When transfections were performed for microscopy studies, a total of one microgram the appropriate DNA plasmids and 3 μl of either PEI for HEK293 cells or GenJet for Huh-7 cells were separately diluted in 25 μl DMEM without serum. They were then mixed and added to each sample after 15 minutes incubation at room temperature.

The cells were fixed with 3.7% paraformaldehyde 24 h after transfection and permeabilized with 0.1% Triton X-100 in phosphate buffered saline (PBS). Primary antibodies were prepared in PBS containing 1% bovine serum albumin (BSA) and incubated for 1 h at room temperature. Secondary antibodies conjugated with fluorophores were prepared in the same buffer but only incubated for 30 min at room temperature. In experiments in which lipid droplets were labelled, bodipy 493/503 at 1 μg/ml in PBS was applied to the samples for 15 minutes at room temperature. The samples were washed three times with PBS before mounting. Immunofluorescence images were taken by using Lecia SP5, Olympus FV1000, or Carl Zeiss LSM5 confocal microscopes equipped with 488, 561 and 633 lasers.

### Electron microscopy

Cells were seeded in 10 cm culture dishes 24 h before processing. If necessary, transfections were pas erformed using the same method for HEK293 cells in immunofluorescence studies except the quantities of DNA plasmids and transfection reagents were increased by 10 times and an additional 24 h was added for protein expression. Specimens were fixed with 2.5% glutaraldehyde EM Grade overnight at 4 °C, followed by 2% osmium tetroxide 1 h at room temperature. Dehydration was accomplished by a series of ethanol solution washes with increasing concentrations until 100% ethanol. They were then infiltrated and embedded with Epon 812 resins using Embed-812 embedding kit following the instructions from manufacturer. Ultra-thin sections were cut by using Lecia ultratome (UCT7) at 70 nm thickness. Images were taken with Hitachi H-7700 Transmission Electron Microscope illuminated with 80 kV electron beam.

### Biochemical fractionation of mitochondria and cytosol

For experiments to detect the Drp1 protein in mitochondria-enriched fractions, HEK293T cells in 150 mm plates were first transfected 10 μg DNA of Myc-NS5A or pMyc empty vector, and at the appropriate time the cells were washed with PBS and harvested in 2 ml buffer containing 0.25 M sucrose, 10 mM HEPES, p.H. 7.4. The cells were homogenized in ice-cold homogenizer by 25 strokes and the homogenates were spun at 600× g for 10 minutes at 4 °C to remove nuclei. The supernatants were further spun at 10,300× g for 15 minutes at 4 °C. The pelleted membranes contained the mitochondria-enriched membrane fraction. The supernatants were spun a few times until no membrane pellet was observed and the resultant supernatants were collected as the cytosol fraction.

### Immunoprecipitation

HEK293T cells were used in experiments shown in Fig. 7, in which the indicated genes were over-expressed. HCV replicon JL 377-2 cells were used in experiments shown in Fig. 4. Cells from 100 mm plates were lysed and scraped in 800 μl of RIPA buffer (20 mM Tris, p.H. 8.0, 100 mM NaCl, 0.1% NP-40, and protease inhibitor cocktail). For experiments in Fig. 4, the RIPA buffer was supplemented with 1 mM of MgCl and 0.1 mM of GDP or GTPγS. After lysis, the cell debris was spun down at 10,000 g for 15 minutes at 4 °C in a micro-centrifuge. 50 μl of the supernatant was taken as input lysate. The remaining 750 μl was subjected to immunoprecipitation using approximately 2 μg of the indicated antibodies and protein A-sepharose beads for at least 4 hours at 4 °C. The beads were washed 3 times before subjected to SDS-PAGE and immunoblotting.

### Statistical analysis

The portion of a mitochondrion defined as tethered by ER was the length of mitochondrial membrane where the distance from ER membrane was less than 20 nm. The quantification method is adopted from the principle of the point-counting method developed by Weibel & Bolender^38^. The perimeters of mitochondria are decorated with evenly-spaced dots using the count tool function in Adobe Photoshop. As the scale and the distance between the dots are known, the length of the circumference of each mitochondrion and the portion tethered by ER can be measured. 60 mitochondria randomly chosen from each specimen were examined and the results were expressed in terms of percentage of mitochondrial circumference tethered by ER. Statistically significant difference between NS5A transfected HEK293 cells and its control or HCV replicon cells and native Huh-7 cells were determined using Student’s *t-* test.

## Additional Information

**How to cite this article**: Siu, G. K. Y. *et al.* Hepatitis C virus NS5A protein cooperates with phosphatidylinositol 4-kinase IIIα to induce mitochondrial fragmentation. *Sci. Rep.* **6**, 23464; doi: 10.1038/srep23464 (2016).
